# Structure of the Plexin Ectodomain Bound by Semaphorin-Mimicking Antibodies

**DOI:** 10.1371/journal.pone.0156719

**Published:** 2016-06-03

**Authors:** Kei Suzuki, Hiroyuki Tsunoda, Ryusuke Omiya, Kyoko Matoba, Takeshi Baba, Sachiyo Suzuki, Hiroaki Segawa, Atsushi Kumanogoh, Kenji Iwasaki, Kunihiro Hattori, Junichi Takagi

**Affiliations:** 1 Laboratory of Protein Synthesis and Expression, Institute for Protein Research, Osaka University, Suita, Osaka, Japan; 2 Research Division, Chugai Pharmaceutical Co. Ltd, Gotemba, Shizuoka, Japan; 3 Department of Respiratory Medicine, Allergy, and Rheumatic Disease, Graduate School of Medicine, Osaka University, Suita, Osaka, Japan; Tulane University, UNITED STATES

## Abstract

Semaphorin family proteins act on cells to mediate both repulsive and attractive guidance via binding to plexin family receptors, thereby playing fundamental roles in the morphogenesis and homeostasis of various tissues. Although semaphorin-plexin signaling is implicated in various diseases and is thus a target of intensive research, our mechanistic understanding of how semaphorins activate plexins on the cell surface is limited. Here, we describe unique anti-plexin-A1 antibodies that can induce a collapsed morphology in mouse dendritic cells as efficiently as the semaphorin 3A (Sema3A) ligand. Precise epitope analysis indicates that these “semaphorin-mimicking” antibodies dimerize cell-surface plexin-A1 by binding to the N-terminal sema domain of the plexin at sites away from the interface used by the Sema3A ligand. Structural analysis of plexin-A1 fragments using negative stain electron microscopy further revealed that this agonistic capacity is closely linked to the location and orientation of antibody binding. In addition, the full-length plexin-A1 ectodomain exhibited a highly curved “C” shape, reinforcing the very unusual dimeric receptor conformation of this protein at the cell surface when engaged with Sema3A or agonistic antibodies.

## Introduction

Plexins constitute a large family of type I transmembrane proteins that serve as the major signaling receptors for the semaphorin family of guidance cue molecules. Although originally discovered as key molecules implicated in the repulsive guidance of neuronal axons [1], the semaphorin-plexin signaling system has since been implicated in a wide variety of physiological processes, including angiogenesis, bone homeostasis, and immune responses [2]. Plexin-mediated semaphorin signaling is initiated by the binding of dimeric semaphorins to the N-terminal sema domain of plexin in the extracellular region, leading to the activation of a complex cascade of reactions in the cytoplasm that modulate the function of the cytoskeleton and cell adhesion machinery [3, 4]. This process results in a change in the morphology and migratory behavior of the cell and induces the typical “collapse” response in signal-receiving cells. The plexin cytoplasmic region consists of two domains—a GTPase activating protein (GAP) domain and a RhoGTPase binding domain (RBD)–that are postulated to work in a concerted manner during semaphorin-induced activation of plexin [2, 5]. However, exactly how the on/off switch of GAP activity is controlled by semaphorin engagement at the ectodomain remains elusive.

Semaphorin 3A (Sema3A) is a prototypic soluble (i.e., secreted) semaphorin that activates A-type plexin (PlxnA) on the cell surface, with the help of its high-affinity co-receptor neuropilin-1 (Nrp-1). In addition to playing an essential role in axon pathfinding during neuronal development, the repulsive signal of Sema3A is responsible for various physiological and pathophysiological events in adult neuronal tissues, such as the suppression of axon regeneration after injury [6]. Sema3A-PlxnA signaling is also critical to the homeostasis of various adult organs. In bones, Sema3A has been reported to function as a coupling factor between bone formation and resorption by promoting and inhibiting the differentiation of osteoblasts and osteoclasts, respectively [7]. This “osteoprotective” effect of Sema3A suggests that modulation of the Sema3A-PlxnA signal may be of potential utility in the treatment of various bone and joint diseases. In the immune system, Sema3A produced by lymphatic endothelial cells directs lymphatic draining of PlxnA1-expressing dendritic cells, enabling effective immune induction by timely antigen presentation [8]. The Sema3A-PlxnA signal has also been shown to exert an immunosuppressive effect by regulating T cell proliferation [9, 10]. On the other hand, the same Sema3A-PlxnA signaling has been reported to enhance the innate immune response by promoting cytokine production in macrophages activated by TLR-agonists [11]. Therefore, both the inhibition and potentiation of this pathway are therapeutically important.

Based on structural analyses conducted on multiple pairs of ligand (semaphorins) and receptor (plexin), it is generally accepted that both soluble and membrane-anchored semaphorins exist as homodimers and induce plexin dimerization on the cell surface by forming 2:2 complexes upon binding [12–14]. However, whether plexin dimerization alone is sufficient for signaling or whether the formation of higher-order networks involving other proteins such as Nrp are prerequisites remains unclear [15]. In the present study, we obtained a panel of monoclonal antibodies (mAbs) that specifically recognize the mouse PlxnA1 ectodomain, among which two have been shown to induce cell collapse by engaging cell surface PlxnA1. This “Sema3A-mimicking” activity was not observed for other mAbs and required the antibody to be divalent, suggesting that the dimerization of cell surface plexin in a specific orientation is required for signaling. Through detailed epitope mapping and negative-stain electron microscopic (EM) imaging of plexin ectodomain fragments, two agonistic antibodies were shown to bind the inner side of the uniquely curved plexin ectodomain, whereas the non-signaling mAb binds the outer side. By combining EM images of the entire PlxnA1 ectodomain, we present a model of the highly unusual dimeric architecture of signal-transducing plexin engaged by Sema3A or agonistic antibodies.

## Materials and Methods

### Expression constructs and protein purification

All PlxnA1 expression constructs were prepared by PCR-based cloning using synthetic DNA corresponding to the human and mouse PlxnA1 genes as templates. The following regions were used to express various truncation ectodomain fragments (using residue numbering based on NP_032907.1); 28–1234 (PlxnA1_1-10_), 28–859 (PlxnA1_1-6_), 28–562 (PlxnA1_1-2_), 28–511 (PlxnA1_1_), 510–1234 (PlxnA1_2-10_), and 860–1234 (PlxnA1_7-10_). The mouse-human chimeric versions and single amino acid mutants of PlxnA1_1-2_ used for epitope mapping experiments were constructed by extension PCR and QuickChange strategies, respectively. Target sequences were inserted after a signal sequence obtained from either artificial protein (HMM+38) [16] or mouse nidogen 1 [17], followed by one of the purification tag sequences chosen from FLAG, Hisx6, and PA-tag [18]. For the versions linked to either a His or a PA tag, a TEV protease recognition site was introduced to enable isolation after purification. The whole ectodomain protein used to make antibodies (containing residues 25–1242) was attached to a FLAG tag and expressed in FreeStyle 293 cells (Life Technologies). The protein was purified from a culture supernatant harvested 5 days post-transfection using anti-FLAG M2 agarose (Sigma) column chromatography followed by Superdex 200 gel filtration chromatography. For the production of PlxnA1 fragments used in EM imaging experiments, His-tagged versions of the expression constructs were used to transfect Expi293F cells (Life Technologies) using an ExpiFectamine 293 Transfection Kit (Life Technologies). Proteins were purified from the culture supernatant using Ni-NTA agarose (QIAGEN), treated with TEV protease to remove the tag segment, and finally purified on a Superdex 200 gel filtration column.

### Generation of PlxnA1-specific recombinant monoclonal antibodies

All animal care and experimental protocols were performed in accordance with the guidelines for the care and use of laboratory animals at Chugai Pharmaceutical Co., Ltd. The protocol was approved by the Institutional Animal Care and Use Committee at Chugai Pharmaceutical Co., Ltd. Five New Zealand White rabbits (Kitayama Labes) were immunized four times with purified FLAG-tagged mouse PlxnA1 ectodomain. Then, the rabbits were humanely sacrificed by exsanguination under anesthesia, and their peripheral blood mononuclear cells and splenocytes were isolated. B cells positive for the production of anti-PlxnA1 were cultivated using the method reported by Seeber et al. [19] and were subjected to sequencing of the genes encoding the antibody variable regions. The DNA segment encoding each rabbit’s Ig variable region was inserted into an expression vector containing the constant region of various IgG species, including rabbit IgG/kappa, mouse IgG1/kappa, mouse IgG2a/kappa, or human IgG4/kappa, to express as recombinant IgG in FreeStyle293 cells. Recombinant IgGs were purified from the culture supernatants using rProtein A-Sepharose (GE Healthcare). Fab fragments were prepared and purified as previously described [20].

### Collapse assay

Dendritic cells were induced from murine bone marrow cells cultured with recombinant mouse GM-CSF (R&D systems) for 7–8 days and were transferred into 96-well flat bottom cell culture plates. After overnight culture, the indicated concentration of anti-PlxnA1 antibodies, anti-Nrp-1 antibody [21], Control-IgG, and/or recombinant murine Semaphorin 3A [22] was added, and the cells were cultured for another 4–5 hours. The cells were fixed with 4% formaldehyde, permeabilized, and stained with Alexa Fluor^®^ 488 phalloidin. After staining, the plates were imaged using a high-content screening system (ArrayScan^®^; Thermo Scientific). Images were taken and analyzed using Cellomics-vHCSTM: Scan software. The morphology of dendritic cells was analyzed using Morphology V4 Algorithms, and the number of cells with a high ObjectConvexHullAreaRatio value (i.e., the ratio of the area of the convex hull of the object to the area of the object) was counted. Due to the very large morphological variation present among the adherent DCs, only 3–14% of the total cells exhibited typical dendritic morphology before stimulation and hence were categorized as “non-collapse cells”.

### Immunoprecipitation and Sandwich ELISA

The epitope of each mAb was first narrowed down to the sema domain using domain deletion mutants and immunoprecipitation. Briefly, DNA coding for the domain-deletion fragments of the mouse PlxnA1 ectodomain shown in S3 Fig was transiently transfected into HEK293T cells using X-tremeGENE HP (Roche), and 1 ml culture supernatants were incubated with 1 μg of anti-FLAG mAb M2 (Sigma, F3165) or mouse-converted anti-PlxnA1 mAbs, together with 10 μl of Protein G Sepharose (GE Healthcare). After incubation at 4°C for 1 h, the beads were washed three times with 20 mM Tris (pH 8.0) containing 150 mM NaCl and were eluted with SDS sample buffer. The bound PlxnA1 fragments were detected by Western blotting using an anti-FLAG rabbit polyclonal antibody (Sigma, F7425) and peroxidase-conjugated anti-rabbit IgG (Sigma, F6154). For detailed mapping, various chimeric versions of PlxnA1_1-2_ fragments C-terminally tagged with a PA tag were used to evaluate reactivity toward each mAb in a sandwich ELISA format. Briefly, fragments were transiently expressed in HEK293T cells using X-tremeGENE HP (Roche) and were captured in microtiter wells coated with the anti-PA tag antibody NZ-1 (Wako Pure Chemical Co., 016–25861). The mouse-converted versions of anti-PlxnA1 mAbs (5 μg/ml) were added to the wells and incubated for 1 h at room temperature, and the bound antibodies were probed with peroxidase-conjugated anti-mouse IgG pre-adsorbed with rat immunoglobulins (Southern Biotech, 1034–05).

### Binding analysis using surface plasmon resonance (SPR)

Protein A/G (PIERCE) was immobilized on the CM4 sensor chip (GE Healthcare) using amino coupling chemistry according to the method provided by the manufacturer. Anti-PlxnA1 mAb solutions (1 μg/ml) were injected at a flow rate of 10 μl/min to achieve capture levels of ~300 RU on the sensor chip. Various concentrations of purified FLAG-tagged PlxnA1 fragment comprising the sema domain (residues 25–511) were injected at a flow rate of 20 μl/min for 2 min to monitor association, followed by dissociation for 3 min. The sensor chip surface was regenerated by injecting Glycine-HCl, pH 1.5, for 30 s, prior to the next cycle of binding analysis. Dissociation constant (*K*_D_) values were obtained by a curve fitting analysis using Biacore T200 Evaluation Software Ver. 2.0. All SPR experiments were performed using a Biacore T200 instrument (GE Healthcare).

### Negative stain electron microscopy and image analysis

For EM imaging of the PlxnA1_1-6_ fragment, approximately 6 μg of purified PlxnA1_1-6_ was incubated with or without an excess of anti-PlxnA1 mAb Fab fragments at 4°C for 30 min and was subjected to gel filtration on a Superdex 200 10/300 GL column equilibrated with 20 mM Tris-HCl, pH 8.0, containing 150 mM NaCl and 2 mM CaCl_2_. The peak fraction obtained from gel filtration was immediately absorbed to glow-discharged (i.e., negatively charged) carbon-coated copper grids prepared essentially as described by Ohi et al. [23] using the hydrophilic treatment device HDT-400 (JEOL, Japan), followed by negative staining with 2.0% (w/v) uranyl acetate. Samples were examined with a Hitachi H-7650 electron microscope operated at 80 kV and a nominal magnification of 80,000×. Images were recorded on a 1,024 × 1,024 CCD camera (TVIPS), corresponding to a resolution of 3.78 Å/pixel. For imaging the larger ectodomain fragment PlxnA1_1-10_, purified protein (~6 μg) was used to prepare specimens in a similar fashion, but they were examined with a JEM-1011 microscope (JEOL) operated at 80 kV and a magnification of 50,000×. Images were recorded on a 4,096 × 4,096 CCD camera (TVIPS), corresponding to a resolution of 2.02 Å/pixel. Image processing procedures included contrast transfer function correlation, particle selection, and 2D classification and averaging, which were performed using EOS [24], EMAN suite [25], and IMAGIC [26], respectively. Particles were selected from individual frames using the program *boxer* in the EMAN suite. The particle images were rotationally and translationally aligned by a multireference alignment procedure and were subjected to classification after specifying 20 (for PlxnA1_1-6_-Fab complex) or 30 (for PlxnA1_1-10_) classes using the IMAGIC program.

### Structural models

A structural model of mouse PlxnA1_1-4_ (containing sema+PSI-1+IPT-1+PSI-2 domains) was prepared using MODELLER [27] from the structure of the corresponding region of mouse PlxnA2 (PDB ID: 3OKT) as a template. For PlxnA1_1-6_ model building, the segment corresponding to the last 2 domains of PlxnA1_1-4_ (IPT-1+PSI-2) were duplicated and appended to the C-terminus to emulate domains 5 (IPT-2) and 6 (PSI-3). To construct a PlxnA1_1-10_ model, four Met IPT-1 structures (PDB ID: 2UZX) were used to supply the missing domains in the PlxnA1_1-6_ model because our own sequence alignment revealed that plexin IPT domains 3–6 are more similar to Met IPT domains than to plexin IPT-1 and IPT-2.

## Results and Discussion

**Fig 1:**
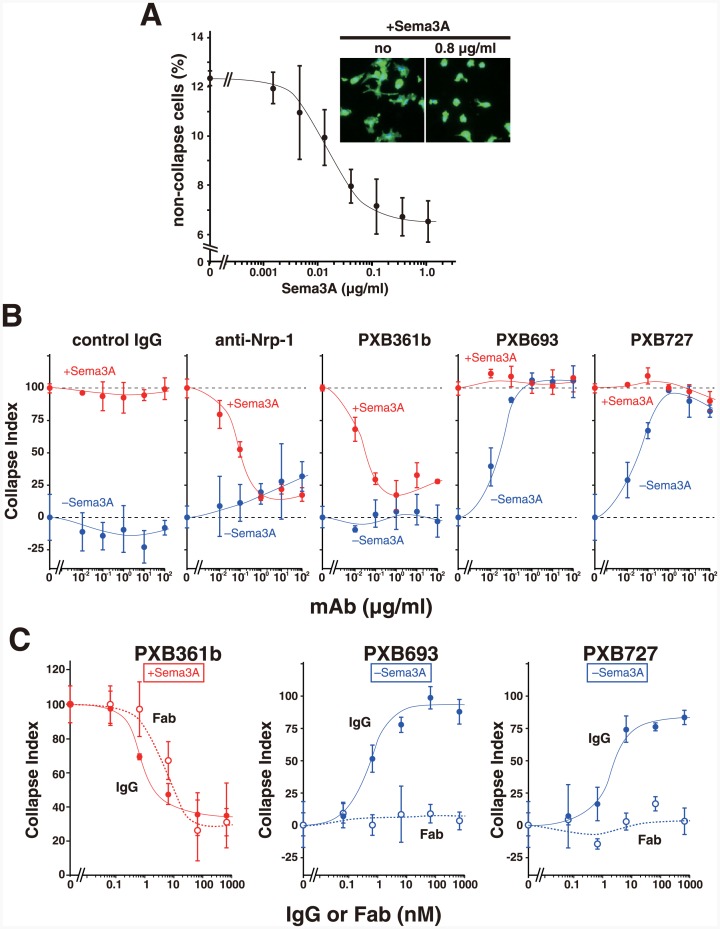
Functional modulation of PlxnA1 by monoclonal antibodies. (A) Sema3A dose dependency. Fluorescent images of adherent DCs (*inset*) w re recorded after incubation with the indicated concentrations of Sema3A, followed by image analysis to assess the collapse morphology of individual cells. The activity was scored as described in the Methods. (B) The effect of anti-PlxnA1 antibodies on Sema3A-induced DC collapse. Various antibodies were added to DCs at the indicated concentrations, together with (red circles) or without (blue circles) 0.8 μg/ml Sema3A, and DC morphology was evaluated after 4–5 h. The collapse response was determined as in (A) and is presented as a “collapse index”, wherein the response obtained with Sema3A was set to 100%. (C) Divalence is required for functional activity of agonistic but not antagonistic antibodies. Three rabbit-mouse chimeric antibodies, in the form of intact IgG (filled circles with solid line) or monovalent Fab fragments (open circles with dotted line), were added to DCs together with (red) or without (blue) 0.8 μg/ml Sema3A, and the collapse response was evaluated as in (B). Antibody concentrations are presented as molar concentrations, assuming molecular mass values of 150 kDa (IgG) and 50 kDa (Fab), respectively. All data are presented as the mean ± SD (n = 3) from one representative experiment out of at least two independent sets of experiments.

It has been shown that Sema3A induces actomyosin contraction in dendritic cells (DCs) to enhance cell motility via binding to cell surface PlxnA1 [8]. We established a method to evaluate the morphological changes of mouse DC (i.e., collapse) induced by Sema3A via image analysis, which revealed the dose-dependent and saturable collapse activity of Sema3A (Fig 1A). This activity can be completely inhibited by a function-blocking antibody against Nrp-1 [21], confirming its dependence on the co-receptor function of Nrp-1 (Fig 1B). Using this assay, we assessed the functional effect of three rabbit anti-PlxnA1 mAbs (Table 1). The mAb PXB361b inhibited Sema3A-induced collapse in a dose-dependent manner, indicating that it is a function-blocking antibody against mouse PlxnA1 (Fig 1B). This inhibitory activity was similarly maintained in the chimeric antibody in which the rabbit Ig constant region was replaced with that of mouse IgG2a (Fig 1C, red closed circles). Furthermore, the Fab fragment prepared from it was also capable of inhibiting Sema3A-induced DC collapse (Fig 1C, red open circles), indicating that no receptor cross-linking is required for this action. In contrast to PXB361b, two other mAbs, PXB693 and PXB727, did not possess this inhibitory activity, even at concentrations as high as 100 μg/ml (Fig 1B, red lines). Instead, they induced the collapsing morphology in DCs in the absence of Sema3A, thereby functioning as “agonists” (Fig 1B, blue lines). The cell collapsing activity of these mAbs was comparable to that of Sema3A, reaching a full response at concentrations >1 μg/ml (= 6.7 nM). Their agonistic activity was unchanged by swapping the Fc region with that of mouse IgG2a or human IgG4 (S1 Fig) but was completely lost when the monovalent Fab form was used (Fig 1C, blue open circles). These results suggest that PXB693 and PXB727 activate PlxnA1 by dimerizing the receptor on the cell surface, with a negligible contribution by the Fc receptors.

**Table 1:** Anti-mouse plexin A1 antibodies.

clone	K_D_ (nM)^a^	property	epitope
PXB361b	6.60 ± 0.04	antagonist	Asn286
PXB693	69.0 ± 1.18	agonist	Ser466
PXB727	5.92 ± 0.03	agonist	Asn482

^a^ Mean ± SD (n = 3) value obtained by SPR experiment.

**Fig 2:**
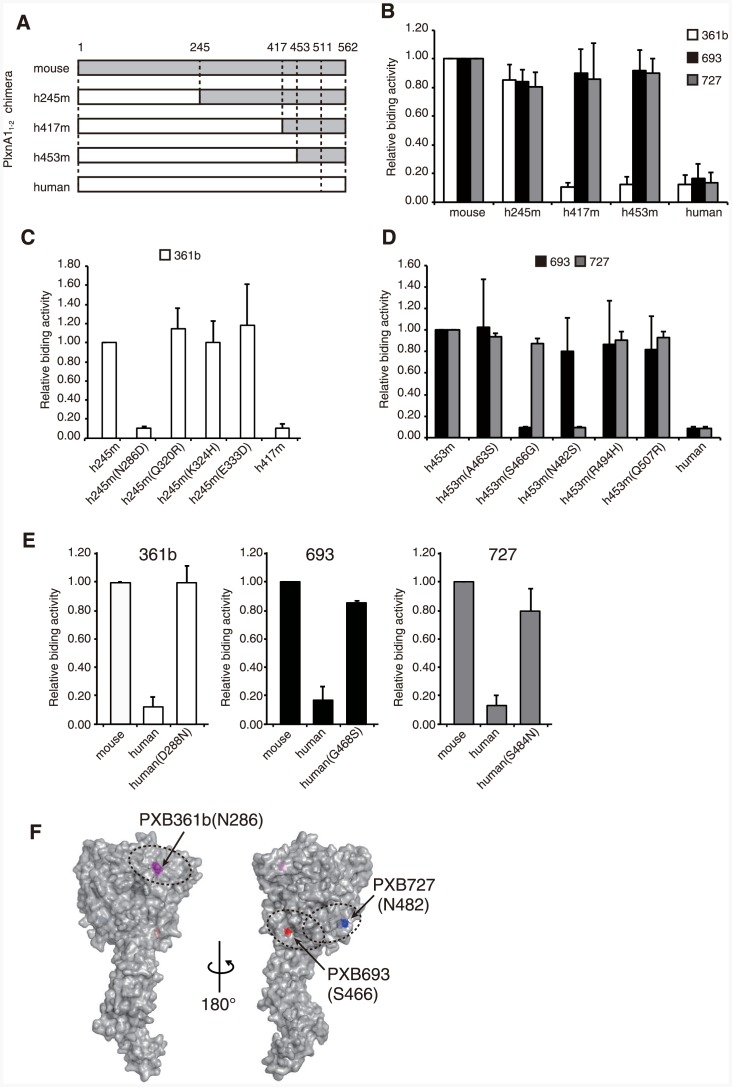
Epitope mapping. (A) Design of human/mouse chimeric PlxnA1_1-2_ used for epitope mapping. (B-E) The reactivity of PXB361b (white), PXB693 (black), and PXB727 (gray) toward various mutant PlxnA1_1-2_ proteins, as assessed by sandwich ELISA. Epitope locations were first roughly determined using the chimeric constructs shown in (A), revealing the requirement of residues 245–417 for PXB361b and 453–508 for PXB693 and PXB727, respectively (B). All mouse-specific residues within these regions were mutated to the human sequence and were subjected to the same assay (C and D). Finally, critical residues were singly mutated to mouse sequence in the context of all-human PlxnA1_1-2_ protein to confirm the gain of reactivity toward each mAb (E). Antibody reactivities are normalized to those obtained with either all-mouse protein (B and E) or the parental chimeras (C and D) and are expressed as the relative value. Data are expressed as the mean ± SD of three independent experiments. (F) A structural model of mouse PlxnA1_1-4_, surface-rendered and viewed from two orientations. The critical epitope residues for each mAb are colored differently, and dotted ovals are drawn around them to represent the estimated size of the antibody-binding footprint.

Although all three mAbs bound mouse PlxnA1 with high affinity (Table 1, S2 Fig) and hence should possess a similar ability to dimerize cell surface PlxnA1, only PXB693 and PXB727 but not PXB361b were able to activate PlxnA1 when added in the absence of Sema3A. This finding strongly suggests that the conformation and/or structure of the forced dimer of PlxnA1 generated by the two agonistic mAbs is signaling-competent, whereas the PXB361b-mediated dimer is not. Such conformation specificity of antibody-induced receptor dimers has been reported in other systems [28]. To gain insight into the structural difference between PlxnA1 bound by different mAbs, we mapped the epitope residue(s) of each mAb. Binding analyses of each mAb using a series of PlxnA1 deletion mutants showed that all three mAbs bind exclusively to the N-terminal sema domain, encompassing residues 28–511 (S3 Fig). However, binding analyses using biolayer interferography revealed that PXB693 and PXB727 compete with each other upon binding to PlxnA1, whereas PXB361b binding was independent of the two agonistic mAbs (data not shown). Although only 18 residues differ between the mouse and human sequences within the 483-residue sema domain (S4 Fig), none of the three mAbs recognize human PlxnA1. We took advantage of this fact and used a chimeric mouse-human PlxnA1 fragment comprised of the sema and PSI-1 domains (PlxnA1_1-2_, Fig 2A) to map epitopes. As shown in Fig 2B, PXB361b lost its binding when residues 245–417 were replaced with human sequence, indicating the location of its epitope within this region. In contrast, the epitope for both PXB693 and PXB727 was shown to be located within residues 453–511 because they bound the shortest fragment containing mouse residues 28–511 (S3 Fig) and all of the chimeric fragments containing the human sequence up to residue 453 (Fig 2A and 2B). By individually mutating species-specific residues within these regions (S4 Fig), critical determinants of recognition by each mAb were identified at the single-residue level to be Asn286 (for PXB361b), Ser466 (for PXB693), and Asn482 (for PXB727) (Fig 2C and 2D). Furthermore, introduction of these mouse residues in the context of human PlxnA1 was sufficient to induce reactivity toward the corresponding mAbs (Fig 2E), confirming their central contribution in the formation of the binding interfaces. When mapped onto the structural model of the mouse PlxnA1 sema domain, their locations were in general agreement with the results of the competition experiment because Ser466 (PXB693 epitope) and Asn482 (PXB727 epitope) are positioned relatively closely to each other, while Asn286 (PXB361b epitope) is located on an opposing surface (Fig 2F).

**Fig 3:**
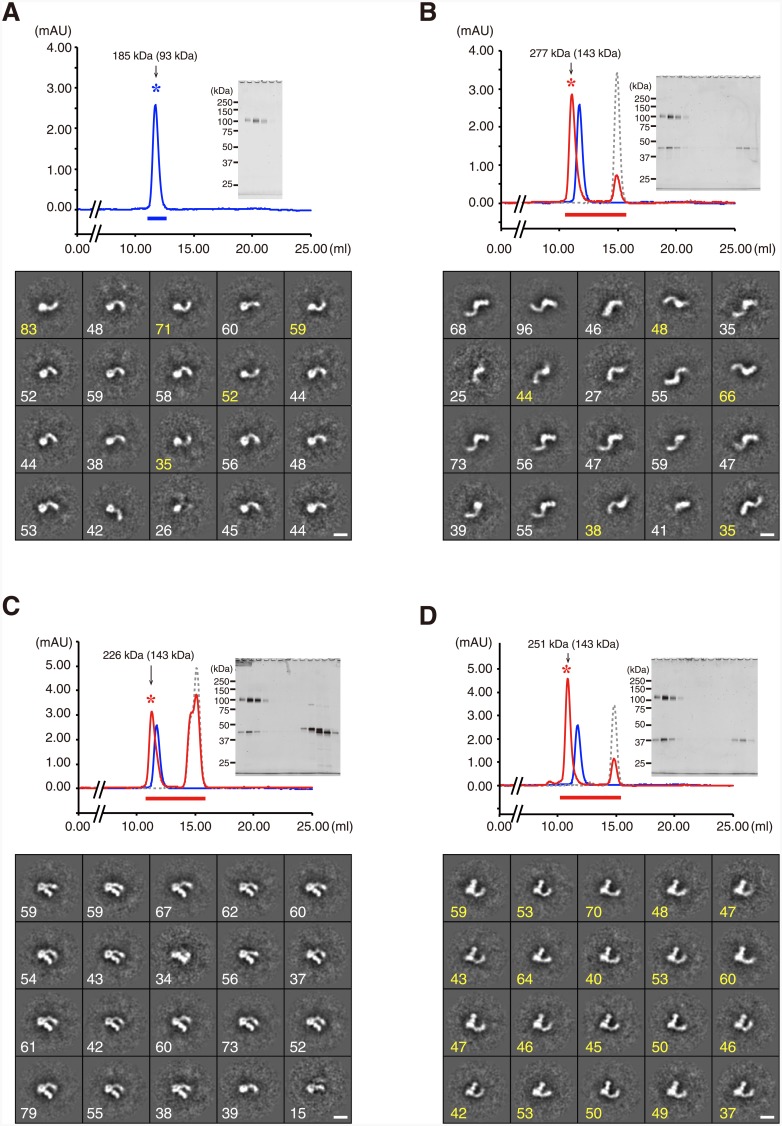
Visualization of the mouse PlxnA1_1-6_-Fab complex using negative-stain EM. Purified PlxnA1_1-6_ protein alone (A) or in complex with Fab fragments derived from PXB361b (B), PXB693 (C), and PXB727 (D) were separated by size exclusion chromatography on a Superdex 200 column. The upper subpanels show the overlaid chromatograms of PlxnA1_1-6_ protein (blue line), PlxnA1_1-6_ mixed with Fab (red line), and Fab alone (gray dotted line). SDS-PAGE gel profiles of the collected fractions (thick horizontal bars) are shown in the *insets*. The peak fraction (asterisk) was subjected to negative-stain EM and image analysis. The molecular size of the collected peak calculated using the positions of standard marker proteins is indicated in the chromatogram, together with the theoretical value in parenthesis. The lower subpanels present a gallery of 20 class averages obtained from ~1,000 selected particles. The number of individual particles represented by each average is shown at the bottom left corner in either white (clockwise) or yellow (counter-clockwise) to indicate their tail curvature orientation. Bar: 100 Å.

**Fig 4:**
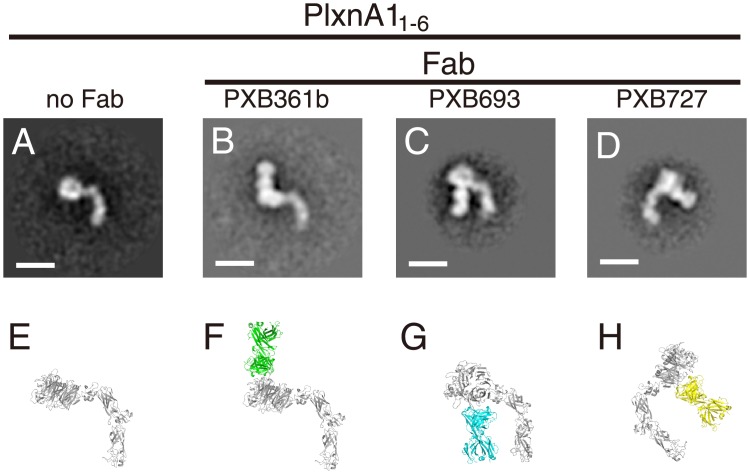
Docking models of the Fab-PlxnA1 complex. (A-D) A representative 2D projection average was derived for each PlxnA1_1-6_-Fab complex by the further refinement of the images shown in Fig 3. Bar: 100 Å. (E-H) Cartoon representation of the modeled PlxnA1_1-6_ protein (gray) upon simulated Fab binding. The viewing orientation for the complex models was arbitrarily chosen to maximize shape similarity with the EM images.

Although epitope mapping was successful for all mAbs at single-residue precision, such information is not sufficient to determine the exact binding footprint on the antigen or the orientation of the antibody Fab arm within the complex. We therefore turned to a negative stain electron microscopy (EM) method to obtain structural information describing the antigen-antibody complexes. The ectodomain fragment PlxnA1_1-6_, spanning domains 1 (sema domain) through 6 (PSI-3 domain) (S3 Fig), was purified by gel filtration chromatography, and the monodispersed peak fraction was subjected to negative staining and visualized under EM (Fig 3A). The raw EM images reveal well-resolved particles with a uniform shape (S5 Fig), enabling the construction of high-quality 2D-averages. The particles exhibited a “comma” shape comprising a globular head attached to a curved tail of 100–120 Å in length (Figs 3A and 4A). This shape is consistent with the expected architecture of the PlxnA1_1-6_ fragment, where the ring-like ß-propeller sema domain is followed by a linear arrangement of alternating PSI and IPT domains (Fig 4E). In particular, the curvature of the first half of the tail, which corresponds roughly to domains 1 through 4), fits well with that observed in the crystal structure of the PlxnA2_1-4_ fragment [12]. The direction of the tail curve was either clockwise/right-handed (70%) or counter-clockwise/left-handed (30%) (Fig 3A), indicating that the adsorption of plexin particles onto the carbon film could occur on either side during EM specimen preparation.

We next conducted the same analysis for PlxnA1_1-6_ complexed with different Fabs. All three Fabs formed stable 1:1 complexes with PlxnA1_1-6_, and the negative-stain EM of the resultant complex isolated by gel filtration gave particle images of a homogeneous shape (Fig 3B–3D and S5 Fig). Importantly, all particles exhibited a dumb-bell-shaped density corresponding to the Fab fragment bound to the globular sema domain. The positions and orientations of the Fab fragments relative to the sema domain were distinct among the three; PXB361b Fab bound the outside of the curvature near the top of the domain, whereas Fabs PXB693 and PXB727 approached from the “inside” at the lower half of the ß-propeller, at slightly different positions (Figs 3B–3D and 4B–4D). Interestingly, the handedness of the plexin tail curvature became uniform upon complex formation with the Fabs of two agonistic mAbs, which differed between PXB693 (clockwise, Fig 3C) and PXB727 (counter-clockwise, Fig 3D). This result indicates that adsorption to the carbon film occurred with opposite preferred orientations for two complexes. In negative-stain EM imaging experiments, antibody Fab fragments appear as either one of two typical shapes; an oval ring (representing the “front” view) or a dumb-bell (representing the “side” view) [29]. The 2D average particle images shown in Fig 4B–4D indicate that all Fabs are viewed from the side, providing information as to the rotational angle of the Fab with respect to the plexin. Using the atomic coordinates of the PlxnA1_1-6_ structural model (Fig 4E), we manually docked a model Fab structure onto the plexin such that the projection views yielded were best fit to the representative 2D averages (Fig 4F–4H). Although the epitope information was not taken into account during docking, the resultant complex models were in perfect agreement with the epitope locations because all three critical residues were buried at the center of the interfaces (S6 Fig). Comparison between the PXB693- and PXB727-bound complex models revealed that two Fabs cannot bind simultaneously, explaining the competitive nature of these mAbs despite the ~30 Å separation of the epitope residues (Fig 2F). Furthermore, the binding footprint of the PXB361b Fab partially overlaps with that of the Sema3A ligand (S6 Fig), indicating that the antagonistic activity of this mAb is due to the direct blockade of ligand binding. We also note that the putative binding site of Nrp-1 suggested by the structure of the Sema3A-PlxnA2-Nrp1 ternary complex [15] lies very close to the position of two agonistic antibodies (S6 Fig, orange), indicating that the binding of Nrp-1 and the antibodies are mutually exclusive.

**Fig 5:**
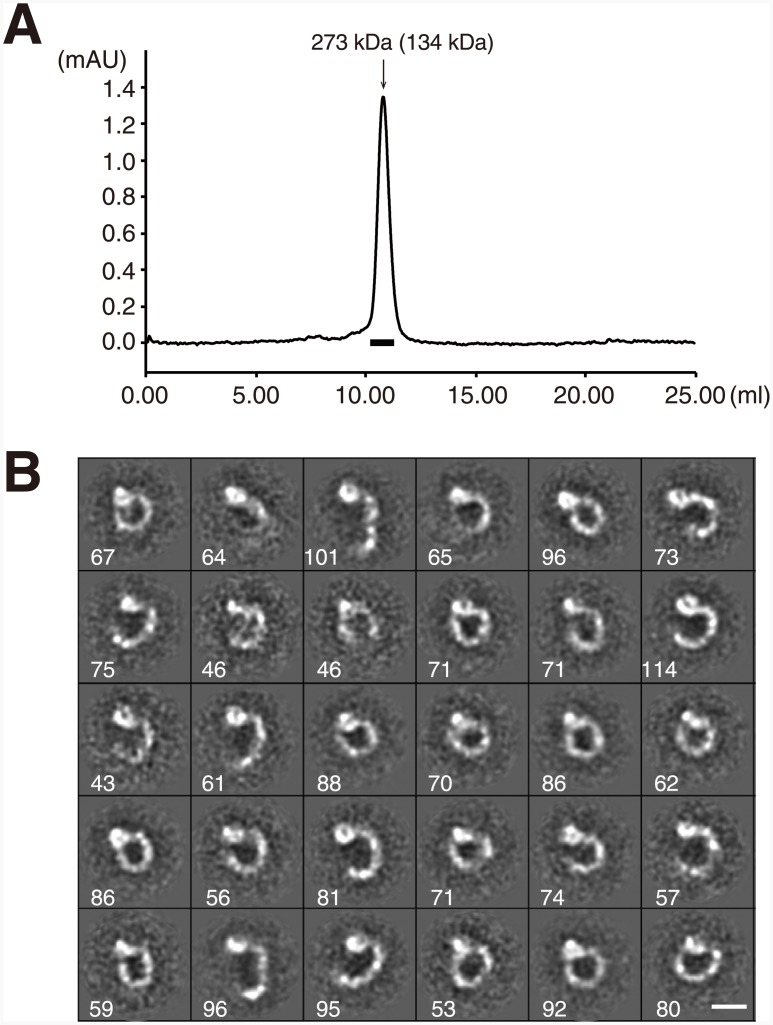
Negative-stain EM visualization of a mouse PlxnA1_1-10_ fragment comprising the entire ectodomain. The full-length PlxnA1 ectodomain fragment was separated by size exclusion chromatography on a Superdex 200 column (A), and the monodispersed peak fraction (thick horizontal bar) was subjected to negative-stain EM and image analysis as in Fig 3. The molecular size of the peak calculated using the positions of standard marker proteins is indicated in the chromatogram, together with the theoretical value in parenthesis. (B) A gallery of 30 class averages was obtained from ~2,200 particles, with the number of individual particles represented by each average shown at the bottom left corner. Bar: 100 Å.

**Fig 6:**
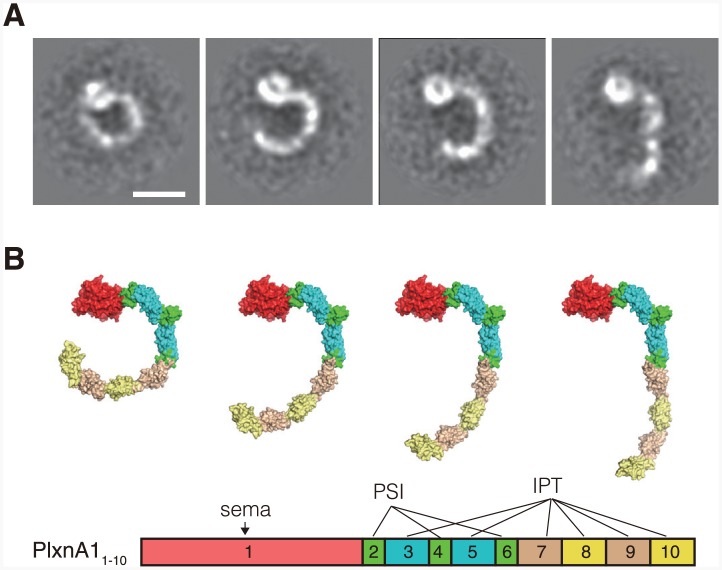
Flexibility of the lower stalk region of the PlxnA1 ectodomain. (A) Four popular class averages for PlxnA1_1-10_ taken from Fig 5. (B) Surface rendering of the structural model of PlxnA1_1-10_. The lower stalk region (i.e., domains 7–10 corresponding to the 3rd to 6th IPT domain) is shown in 4 different orientations relative to the upper portion. The domains are color-coded as indicated by the schematic shown at the bottom.

To determine the conformation of the plexin receptors on the cell surface, we next imaged a full-length ectodomain fragment of PlxnA1 comprising domains 1 through 10 (PlxnA1_1-10_). Mouse PlxnA1_1-10_ protein eluted as a single peak corresponding to a size of 273 kDa in gel filtration experiments (Fig 5A), and this peak was comprised of particles with ring or “C” shapes when visualized by negative-stain EM (S7 Fig). 2D averaging of the particle images derived many classes with distinct but similar shapes. Importantly, essentially all particles exhibited a globular density corresponding to the sema domain, with a curved stalk projecting in a clockwise direction that was consistent with the domain organization of the PlxnA1_1-10_ monomer (Fig 5B). Although the apparent molecular size calculated from the elution position based on gel filtration was approximately 2 times larger than the theoretical value (134 kDa), we could not identify any particles that were consistent with a dimer configuration. The disproportionately large Stokes radius exhibited by PlxnA1_1-10_ is thus likely to have originated from its unique shape rather than from dimerization. In most class averages, the stalk region exhibited several distinct densities that likely correspond to ~100-residue IPT domains, although the last (i.e., IPT-6) domain tended to disappear in the averages (Fig 5B). The curvature of the stalk was not uniform, resulting in a variety of overall shapes ranging from a nearly closed ring to a wide-open C shape (Fig 6A). However, comparison with the images of PlxnA1_1-6_ (Fig 4A) revealed that this variation was primarily due to the pivoting movements of the lower half of the stalk (domains 7 through 10, corresponding to IPT-3 through -6) with respect to the upper half, with the domain 6–7 boundary serving as the hinge point. In fact, appending four consecutive IPT domain models after the PlxnA1_1-6_ model at different angles produced models of the entire plexin ectodomain that exhibit excellent similarity to the representative class averages (Fig 6B). Because we did not identify any straight or S-shaped particles on the grid, the interdomain mobility of the stalk seems to be limited to one direction. Furthermore, this C-shaped ectodomain architecture was observed for not only mouse PlxnA1 but also human PlxnA1 and human PlxnD1 ectodomains (data not shown), suggesting that it is a common structural feature shared among plexin receptors.

**Fig 7:**
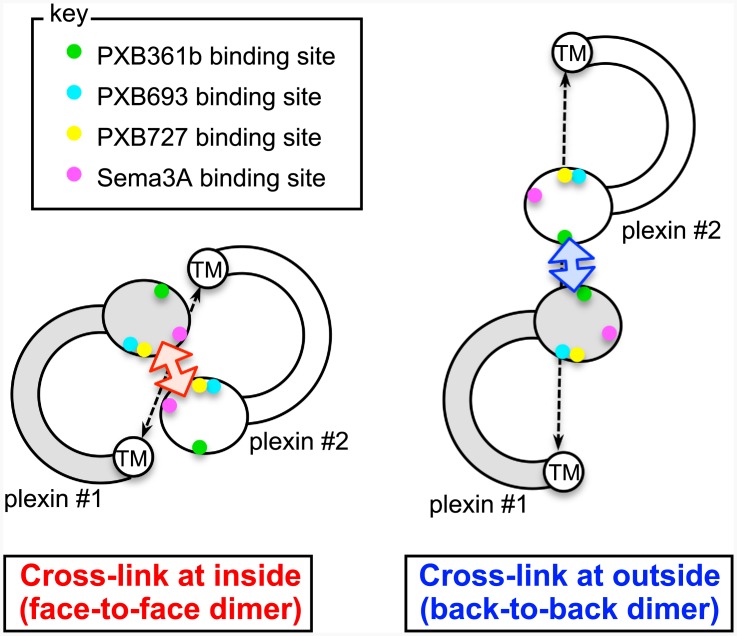
Hypothetical arrangement of the cell-surface plexin dimer. C-shaped plexin ectodomains are schematically drawn in this bird’s eye view (i.e., looking down from above the cell). When two plexin heads (oval) are cross-linked from inside the curvature by either semaphorin or mAb in a face-to-face fashion (left), their TM domain is free to approach, leading to the formation of a compact and presumably productive signaling complex. In contrast, dimerization in a back-to-back orientation (right) leads to a wide separation between the two TM (and hence the following GAP) domains. Binding site locations for antibodies and the semaphorin ligand within the sema domain are indicated by differently colored dots (see the *key* description).

The structural information about plexin receptors available thus far, including EM, crystal, and model structures, were combined to simulate signaling events on the cell surface. If we simply place the C-shaped plexin ectodomain on the cell surface, assuming that the stalk base will emanate from the membrane in a vertical manner, there is a steric clash between the bowed N-terminal sema domain and the membrane. Therefore, we speculate that the stalk base and the following membrane-spanning domain possess a high crossing angle with the membrane and/or contain a severe kink, allowing the full-length ectodomain of plexin to lie near the membrane. Zhang et al. have performed molecular dynamics simulations of the TM domain of A-type plexins on an Anton machine and have reported that these plexins tend to be severely kinked at the N-terminal region [30]. This unusual arrangement of the plexin ectodomain is depicted schematically in Fig 7, which represents the projected molecular shape in a bird’s eye view (i.e., looked down on from the top). The consequence of the C-shape ectodomain lying nearly parallel to the membrane in this model is most striking when we simulate receptor cross-linking. If two sema domains are cross-linked by a bivalent agonistic antibody (e.g., PXB693 or PXB727) or by a semaphorin dimer binding at the inner side of the curvature, then the resulting complex will be a compact face-to-face dimer (Fig 7, left), whereas cross-linking by “outside binders” such as PXB361b will result in an elongated back-to-back dimer (Fig 7, right). These two dimers considerably differ in the distance between the two transmembrane domains (dashed lines), which directly relates to the distance between the cytoplasmic GAP domains. The putative “signal-producing” compact dimer configuration is much more likely to facilitate direct interaction between the cytoplasmic GAP domains, which is in general agreement with the idea that the plexin GAP is activated upon dimerization [31]. Based on these considerations, we propose that anti-plexin mAbs mimic the action of semaphorin ligands only when they cross-link plexins in a face-to-face fashion. In contrast to the semaphorin dimer, which can serve as a rigid dimerizing scaffold, two Fab portions in the IgG are connected by a flexible hinge and can be widely separated. Therefore, antibodies may induce unique dimer configurations that are similar but distinct from the semaphorin-induced physiological dimer, as reported for agonistic anti-EpoR antibodies [32].

The semaphorin-plexin signaling axis has been implicated in a wide variety of physiological and pathophysiological processes and is known to regulate cell behavior in both positive and negative ways. Therefore, both the inhibition and activation of this system have great therapeutic potential, and the present study proves that efforts to develop antibodies targeted at semaphorin-plexin systems are of great value. Furthermore, structural and functional analyses of ligand-mimetic antibodies led to a new hypothesis regarding the conformational mechanism of plexin activation. However, we do not possess sufficient structural understanding of physiological plexin activation on cell surface. For example, compelling evidence exists that the plexin GAP domain can be activated by dimerization through the parallel association of each transmembrane helix [31], but such a configuration is difficult to reconcile with the model shown in Fig 7, which requires a heavily tilted transmembrane helix. In this regard, it is important to note that plexin transmembrane domains may undergo significant conformational changes and/or exchanging transitions between various oligomeric states, as suggested by computational simulations [30]. One plausible scenario is that a complex network of molecular interactions on both the extracellular and intracellular sides involving multiple membrane and cytoplasmic proteins may reorganize the semaphorin (or antibody)-induced primary dimer of plexins, leading to the formation of the higher-order signaling platform required for a full response, as proposed by several studies [2, 33]. Sophisticated antibody engineering combined with an accurate mechanistic understanding of receptor activation can result in the development of better agonistic antibodies [28, 34]. Thus, determining the high-resolution structures of signaling-competent full-length plexin and the development of mAbs capable of modulating the functions of various plexins are eagerly awaited.

## Supporting Information

S1 Fig Agonistic activity of PXB693 and PXB727 carrying various constant regions. Two rabbit anti-PlxnA1 mAbs with agonistic activity were converted to a different subclass/species by replacing the constant regions of rabbit sequence (purple) with mouse IgG2a (green) or human IgG4 (light blue). Their effects on DC morphology in the presence (red circles) or absence (blue circles) of 0.8 μg/ml Sema3A were evaluated and are shown in Fig 1. Data are presented as the mean ± SD (n = 3) of a representative experiment.
(EPS)

S2 Fig Affinity measurement of three anti-PlxnA1 mAbs. Various concentrations of mouse PlxnA1_1_ were flowed over a sensor chip surface immobilized with the indicated anti-PlxnA1 mAbs via Protein A/G. Shown are representative sets of sensorgrams after subtracting the control curve, overlayed with global fitting curves (black dashed lines) obtained using Biacore T200 evaluation software, version 2.0.
(PDF)

S3 Fig The epitopes of three anti-PlxnA1 mAbs are localized within the sema domain (i.e., domain 1). (A) A schematic presentation of domain-deleted PlxnA1 ectodomain constructs. (B) An assessment of the reactivity of each mAb toward various truncated PlxnA1 fragments by immunoprecipitation. Note that the FLAG antibody can immunoprecipitate all constructs, confirming their successful expression and secretion into the culture medium.
(EPS)

S4 Fig Sequence alignment of the mouse versus human PlxnA1 sema domain. Amino acid sequence corresponding to the sema domain of mouse (NP_032907.1) and human (NP_115618.3) PlxnA1 are shown, with species-specific residues and signal sequences shown in yellow highlighting and gray italic letters, respectively. Residue numbers for the boundaries used in the design of chimeric constructs (black) as well as the key epitope residues for PXB361b (N286, magenta), PXB693 (S466, red), and PXB727 (N482, blue) are indicated above the alignment.
(EPS)

S5 Fig Representative EM field views of a negatively stained mouse PlxnA1_1-6_ fragment-Fab complex. (A) PlxnA1_1-6_ alone, (B) PlxnA1_1-6_ in complex with PXB361b Fab, (C) PlxnA1_1-6_ in complex with PXB693 Fab, and (D) PlxnA1_1-6_ in complex with PXB727 Fab. Some views containing large tobacco mosaic virus (TMV) particles were used as internal pixel size calibration standards as well as to monitor the quality of the grids.
(EPS)

S6 Fig Localization of the binding interfaces. A structural model of mouse PlxnA1_1-4_ (gray surface model) is shown with simulated binding of three Fabs (Fig 4) and experimentally determined binding of the Sema3A ligand (light pink surface, taken from PDB ID: 4GZA), viewed from three different orientations. The putative binding surface of the a1 domain of Nrp-1, as suggested by the Sema3A-PlxnA2-Nrp1 ternary complex structure (PDB ID: 4GZA), is also shown in orange.
(PDF)

S7 Fig Representative EM field views of a negatively stained mouse PlxnA1_1-10_ fragment. (EPS)
