# Supplementary figures and images for: Structure of the Plexin Ectodomain Bound by Semaphorin-Mimicking Antibodies
Source: PLoS One. 2016 Jun 3;11(6):e0156719. doi: 10.1371/journal.pone.0156719 (PMC4892512; doi:10.1371/journal.pone.0156719)

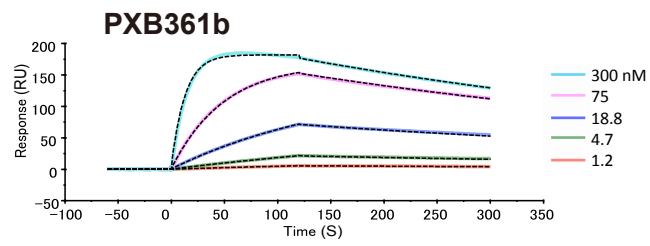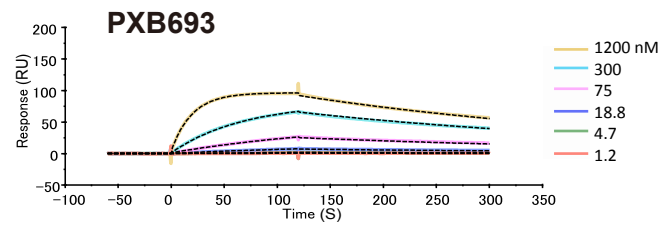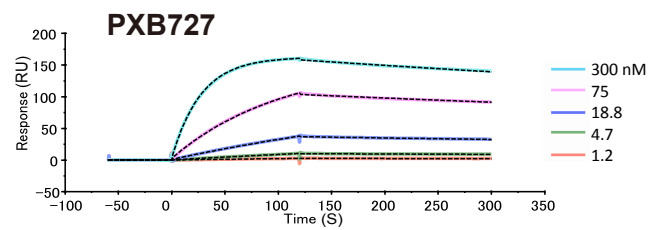

S2 Fig

Supplement: S2 Fig — Various concentrations of mouse PlxnA11 were flowed over a sensor chip surface immobilized with the indicated anti-PlxnA1 mAbs via Protein A/G. Shown are representative sets of sensorgrams after subtracting the control curve, overlayed with global fitting curves (black dashed lines) obtained using Biacore T200 evaluation software, version 2.0. (PDF) [file pone.0156719.s002.pdf]

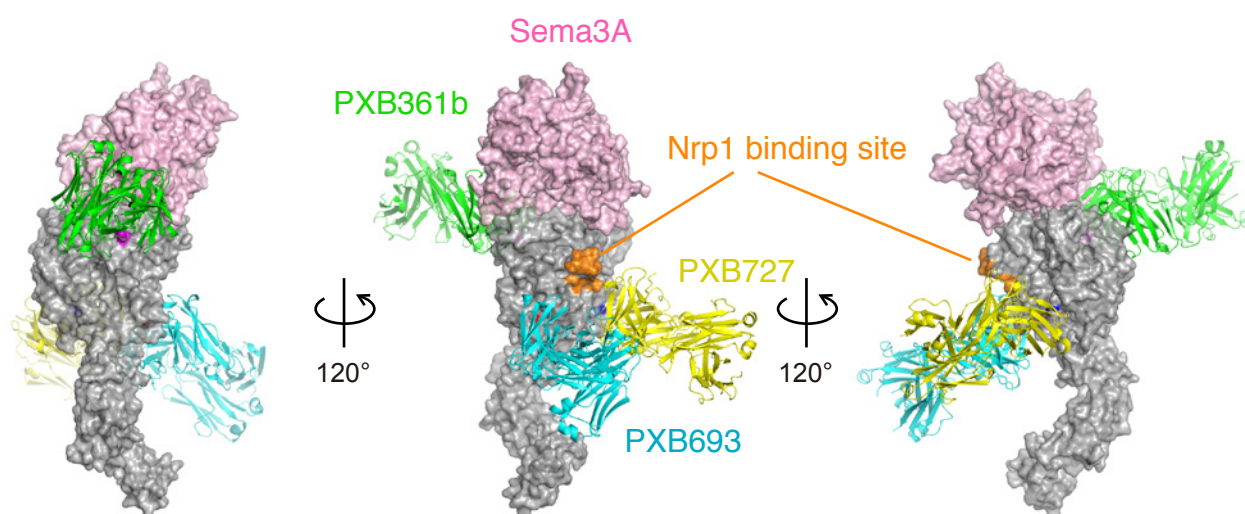

S6 Fig

Supplement: S6 Fig — A structural model of mouse PlxnA11-4 (gray surface model) is shown with simulated binding of three Fabs (Fig 4) and experimentally determined binding of the Sema3A ligand (light pink surface, taken from PDB ID: 4GZA), viewed from three different orientations. The putative binding surface of the a1 domain of Nrp-1, as suggested by the Sema3A-PlxnA2-Nrp1 ternary complex structure (PDB ID: 4GZA), is also shown in orange. (PDF) [file pone.0156719.s006.pdf]
